# Pharmaceutical Application of Process Understanding and Optimization Techniques: A Review on the Continuous Twin-Screw Wet Granulation

**DOI:** 10.3390/biomedicines11071923

**Published:** 2023-07-06

**Authors:** Jie Zhao, Geng Tian, Haibin Qu

**Affiliations:** Pharmaceutical Informatics Institute, College of Pharmaceutical Sciences, Zhejiang University, Hangzhou 310058, China; zhaojie_1021@zju.edu.cn (J.Z.); iamtiangeng@zju.edu.cn (G.T.)

**Keywords:** twin-screw wet granulation (TSWG), process understanding, process model, continuous manufacturing (CM), process analytical technology (PAT), quality control

## Abstract

Twin-screw wet granulation (TSWG) is a method of continuous pharmaceutical manufacturing and a potential alternative method to batch granulation processes. It has attracted more and more interest nowadays due to its high efficiency, robustness, and applications. To improve both the product quality and process efficiency, the process understanding is critical. This article reviews the recent work in process understanding and optimization for TSWG. Various aspects of the progress in TSWG like process model construction, process monitoring method development, and the strategy of process control for TSWG have been thoroughly analyzed and discussed. The process modeling technique including the empirical model, the mechanistic model, and the hybrid model in the TSWG process are presented to increase the knowledge of the granulation process, and the influence of process parameters involved in granulation process on granule properties by experimental study are highlighted. The study analyzed several process monitoring tools and the associated technologies used to monitor granule attributes. In addition, control strategies based on process analytical technology (PAT) are presented as a reference to enhance product quality and ensure the applicability and capability of continuous manufacturing (CM) processes. Furthermore, this article aims to review the current research progress in an effort to make recommendations for further research in process understanding and development of TSWG.

## 1. Introduction

In the past decade, continuous manufacturing (CM) of pharmaceutical solid dosage forms has gained significant attention [1]. As an emerging technology, it has high potential to improve their product quality and reliability, reduce the manufacturing costs and waste, and increase manufacturing flexibility and agility in response to fluctuations in drug product demand [2]. The release of the “Continuous Manufacturing of Drug Substances and Drug Products, Q13” by the ICH, offers further evidence of the ongoing efforts of regulatory bodies to support the modernization of the pharmaceutical industry throughout the development and implementation of innovative continuous approaches.

Recently, twin-screw wet granulation (TSWG), as a typical application in continuous pharmaceutical industry, has gained significant attention. Granulation is an important process of solid dosage drug manufacturing. It could improve the properties of granule, such as particle size, dissolution rate, bulk density, powder flowability, compressibility, active pharmaceutical ingredient (API) uniformity, granular strength and density by wet granulation or dry granulation method [3,4]. Compared with the batch granulation techniques, such as the fluid-bed granulation, roller compaction, and high-shear wet granulation methods, TSWG could be seen as a promising tool for transforming traditional batch mode to CM in the pharmaceutical industry [5]. It has the advantages of processing high volume, higher production efficiency, short residence time, and better mixing and controlling processes [6]. It can be readily integrated into the CM of pharmaceutical dosage forms. In addition, TSWG provides easier process scale-up, better quality assurance, low production costs, and materials waste [7].

A deep understanding of the process is essential for the CM implementation of pharmaceutical products. Unlike traditional batch manufacturing, CM involves a series of integrated and interconnected unit operations that work together to produce a consistent quality product. In batch processes, material quality is assumed to be uniform throughout the entire batch, while material quality is subjected to variations in continuous processes if the process is not within a state of control [8]. The Quality by Design (QbD) guidelines promote the thorough generation of product and process understanding, which is necessary for the continuous and systematic operation of processes and quality control of products. This includes determining essential critical quality attributes (CQA), critical process parameters (CPP), and control strategy [9]. In the conventional batch production mode, the product quality control is carried out by sampling and analyzing after each unit step process, which is not suitable for the continuous process. With the development of advanced process analytical technology (PAT), it offers a path towards the successful deployment to maintenance a state of control for CM during production and allow real-time evaluation of system performance [10]. Moreover, the methods for monitoring, controlling, and optimizing in CM necessitate distinctive strategies. A QbD approach in conjunction with PAT has thus been identified as a promising pathway towards the successful industrial implementation of CM for pharmaceutical uses [11].

However, the technology transfer from batch granulation to continuous granulation based on TSWG is considered a challenge with technical difficulty due to its flexible geometry structure, diverse formulations, and complex granulation mechanism. At the same time, the product produced by TSWG needs to be conducive to the downstream processing, and the granule produced by TSWG need to meet the high regulatory standards in the pharmaceutical industry to ensure the safety and quality of the products. Facing the considerable demand of TSWG in CM for drugs, enhancing the knowledge of manufacturing processes and improving the control of the TSWG process are the key to establishing adaptive processing procedure for TSWG. Therefore, this work focuses on the process understanding for TSWG, discusses the recent progress in process understanding and optimization for TSWG, including some progress in process model construction, the method for process monitoring, and the progress of process control for TSWG. This article aims to enhance comprehension and advancement of processing by reviewing and presenting existing research on twin-screw granulation, which is gaining interest, and the crucial need for viable continuous processing.

## 2. Process Understanding and Optimization

**Figure 1:**
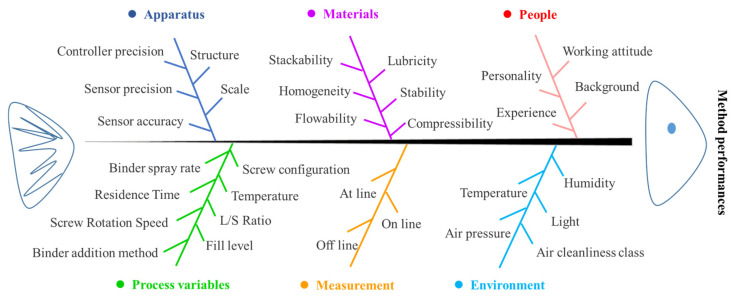
Ishikawa diagram for the twin-screw granulation process behavior.

Understanding the process is the first step for the application of TSWG. There are many factors that affect the TSWG process, which can be divided into six categories, i.e., apparatus, materials, people, process variables, measurement, and environment, as shown in Figure 1. The variation of variables makes the TSWG system an exceptionally large operating space. Therefore, because of the complexity and variability of the TSWG system, it is required a systematic understanding of the dynamic change in the characteristics of the material while progressing in the TSWG process to control. The process model development approach can be adopted through the statistical, mechanistic, and hybrid models in the TSWG process to increase the knowledge of the granulation process. The process design, control, and variable optimization of the TSWG process can also be developed by modeling techniques.

### 2.1. Empirical Model

Empirical models are mathematical expressions that are created by analyzing observed data from experiments or observations. Empirical models are often used in scientific research to quantify relationships between variables in a specific context or system. Using the QbD principle, the empirical model developed based on the statistical data from design of the experiment (DoE) can evaluate the effect of CPP and critical material attributes (CMA) on the CQA simultaneously. The statistical data from the DoE could contribute to developing the model and defining the design space, thus ensuring desired quality of the product [12]. It is an effective method applied to TSWG for process understanding, designing, controlling, and optimizing continuous pharmaceutical manufacturing.

Studying the process settings and generalizing the conclusions contribute to better understanding of the TSWG. Many researchers have used the QbD principle to study the TSWG process. At present, the research on CPP in the TSWG process mainly focuses on the geometry and configuration of the screw elements, process variables, and product quality. Different experimental design methods have been performed to evaluate the influence of material properties (formulation, adhesive), process variables (type and number of screw elements, screw speed, liquid feed rate, and liquid to solid (L/S) ratio) on the characteristics of granules [1,13,14,15,16,17]. Seem et al. [18] summarized the comprehensive review of the experimental twin-screw granulator (TSG) literature, indicating the complex interplay between the role of screw element type, screw configuration, feed formulation and liquid flowrates on the granules. The specific parameters haven been investigated, as well as the CQA of granules defined in the corresponding paper, and the DoE method used to process development with corresponding modeling methods have been summarized in Table 1. The overview of research papers that used the QbD principle for process understanding and optimization on TSWG can make a reference for further research.

The screw configuration, screw speed, and throughput have a large impact on the granule properties. The degree of shear applied to the material during granulation process can be varied by adjusting of these parameters. The screw configuration includes the pitch and length of the conveying elements, the thickness and angle of the kneading elements. The process variables consist of the properties of the materials and binder, L/S ratio, screw speed, and material feed rates, etc. Due to the large diversity of the model formulations and equipment, it is difficult to draw general conclusions. However, based on the literature, the results of this research showed that the process parameters, such as L/S ratio, screw speed, and throughput, are the key process variables of the TSWG process. It is important for the process understanding to clarify the influence mechanism of the process conditions on the particle properties.

The L/S ratio has been extensively identified as the most predominant factor in preparing TSWG products with desired quality attributes [36,37]. It is reported that the low L/S ratio produces the granules with broad and bimodal size distribution, while the size distribution becomes narrow and monomodal at high L/S ratio that are too large to be directly used for tableting [18]. The increase of L/S ratio could promote the growth of granules, increase bulk and tapped density, and facilitate the strength and flow properties of the particles. A second-order polynomial model based on a central composite face-centered experimental design demonstrates that as the L/S ratio increases from 10% to 70%, the proportion of fines dramatically decreases due to the high aggregation rate resulting from the liquid [23]. Moreover, the initial multi-factor analysis of variance (ANOVA) based on a face-centered cubic design model indicated that bulk and tapped densities and the shape of granules were greatly affected by the L/S ratio [22].

Changes in throughput can impact the barrel filling, with a higher value leading to more material hold-up volume due to the compression and packing of primary particles. A PLS model that was built based on a full factorial experimental design showed that the increment of material throughput could quickly lower the yield due to insufficient mixing between powder and liquid [21]. A non-linear quadratic mathematical model based on a Box–Behnken experimental design showed that the increased throughput could produce granules with a narrower PSD by reducing the oversized granules and fines [14]. Finally, a stepwise least squares regression model built on a sequential experiment showed that throughput significantly influences particle porosity. The larger porosity could be largely due to the high upstream throughput force and reduced residence time, the materials were conveyed faster, and particles suffered from less interaction and compaction [27].

### 2.2. Mechanistic Model

**Figure 2:**
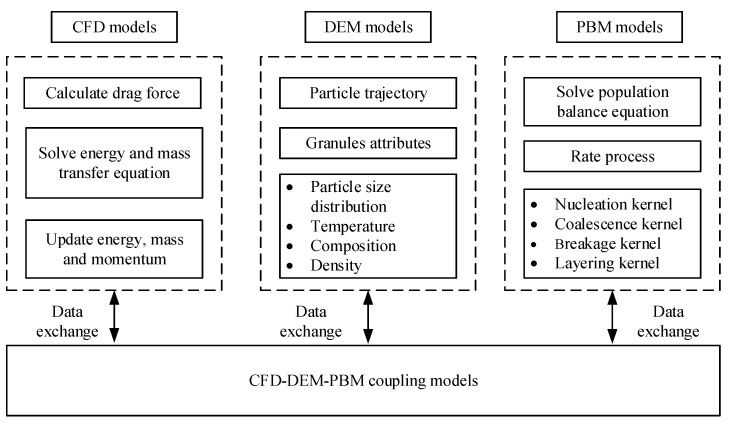
Schematic of the models for granulation.

Mechanistic models can be constructed to reveal the mechanism of material transformation in the TSWG process, and the model could then be coupled with process control systems to allow control of product specification in TSWG systems. Among the various modeling and simulation methods applicable for the wet granulation process, the population balance model (PBM), discrete element method (DEM), computational fluid dynamics (CFD), and the combination of these models have drawn widespread attention (Figure 2).

DEM, as a scientifically meaningful model method, is playing an increasingly important role in simulating a variety of phenomena including the mechanistic aggregation, consolidation, and breakage rate expressions in the TSWG process. DEM adopts Newton’s second law and can track the spatial coordinate of each particle. It can obtain compartmental residence time data, particle velocities, collision rates, and provide macroscopic information and microscopic insights into the complex TSWG process [38]. With the improvement of high-performance computer capabilities such as the graphics processor unit (GPU) acceleration and parallel computing, the DEM can speed up the simulation and overcome the problem of computations further, indicating that the real-time simulations become possible for the TSWG implications in the near future.

PBM employs the principle of particle number density conservation and can simulate the granulation process of particles in batch or continuous production mode. Moreover, it can track the particle properties in the granulation process. Different dimensional PBM models have been developed for process simulation, such as 1D PBM [39] and multi-dimensional PBM [40]. The advanced modeling tool can predict granule size, PSD, liquid content distributions, RTD, etc. [41,42,43]. Based on the PBM framework, the model was also constantly improved. For instance, in improving a 1D PBM [37,44] for process simulation, a high-dimensional stochastic PBM was constructed to estimate the residence times for different screw element geometry [45]. A novel four-dimensional, stochastic PBM for twin-screw granulation was proposed to describe the mechanistic rates and track more complex particle properties and their transformations [46].

Besides the commonly used mechanistic models, a regime map based on the regime theory can present a method to describe the variation and behavior in the granulation process [47]. Moreover, this semi-mechanistic technique can predict the RTD curve [48] and mean residence time (MRT) with decent precision for TSWG.

### 2.3. Hybrid Model

Compared with the complex numerical simulation models, easier and more efficient modeling methods are an urgent requirement for the simulation application of the TSWG process. The hybrid model combined multiple modeling technique can provide a more comprehensive understanding of the complex pharmaceutical processes, which is an effective approach to improve the limitations of a single kind model. Artificial neural network (ANN) model provides a powerful tool for data analysis and pattern recognition for drug development processes. However, the ANN model has a complex model structure, the network involves input layer, hidden layer(s), and output layer. Many parameters need to be optimized to achieve better model performance. To improve the predictive capability of the artificial neural network (ANN) model, kriging interpolation is applied to get new data to develop an improved ANN model for the mean residence time. The results showed that the hybrid model combined with kriging interpolation could predict the mean residence time with more accuracy for TSWG [49].

In order to obtain a more accurate model, the models combining CFD, DEM, and PBM have also been proposed. A multi-scale, compartmental PBM-DEM model of a continuous TSWG process was presented [50]. The PBM describes the mechanistic rate aggregation, breakage, consolidation, and the DEM was used to obtain residence time information and gather collision and velocity information. The simulated results are consistent with experimental trends, demonstrating the model’s qualitative ability to model and predict the effects of screw element configuration and process parameters on the product size distribution, porosity, and liquid distribution.

A new framework based on fuzzy logic is proposed to predict the granule size distribution accurately in the TSG process, outperforming the standard fuzzy logic systems and the ANN [51]. A versatile simulation model with high-performance for TSWG can be further used for defining the design space. A hybrid model combining PBM and ANN was developed, which was utilized to determine particle size distribution and mean residence time, respectively [36]. In addition, the model can simulate and predict the PSD with fairly high accuracy throughout the simulations, as well as define the design space to develop and optimize the TSWG process.

## 3. Process Monitoring

The purpose of using PAT tools in the TSWG process is to monitor, control, and optimize the process parameters to produce the granule with desired quality. Real-time monitoring of the manufacturing process can help to visualize and provide the product information throughout the process, and with the real-time in-process measurement of CQAs, the off-spec products can be effectively identified and removed from the stream to ensure safety and quality. The common object of granulation technology is to prepare granules with a fine appearance, excellent uniformity, and high yield. Review of the published papers, qualitative and quantitative analysis using PAT tools for granule produced by TSWG have been reported. These analyses include the API content of the granules, RTD, moisture content, PSD, and particle density, etc. The powder properties of particles are complex, the more parameters detected during the process, the more information represented. However, as the number of detection parameters increases, the testing time or the duration of data processing will also increase, ultimately leading to a reduction in detection efficiency. In specific applications, it is necessary to consider comprehensively the experimental conditions and the operability, thus to select the parameters that affect drug quality as much as possible in the TSWG process.

There have been many reports on the application of PAT tools in process monitoring of TSWG, incorporating techniques such as near-infrared (NIR) spectroscopy, Raman spectroscopy, spatial filter velocimetry (SFV), etc. These techniques can contribute to the enhancement of product quality through the development of process knowledge. Moreover, PAT tools are an indispensable element in the implementation of process control strategies [52].

### 3.1. Spectroscopy

A molecular vibrational spectroscopic technique like NIR spectroscopy and Raman spectroscopy techniques enhanced real-time process monitoring application efficiency. The qualitative and quantitative analysis with non-invasive and non-destructive measurement can be easily used for continuous process flow to obtain multi-dimensional information online.

The NIR spectrum contains both the physical and chemical characteristics of the material with specific absorption and scattering effects. Hence, it is specific and sensitive to the particle properties (e.g., particle size and distribution, surface morphology, density, shape, and surface texture) that affect the path length and light penetration [53]. Therefore, the influence of different installation positions of the NIR spectroscopy probe on in-line measurements was investigated. By characterizing NIR interfaces in different positions of a TSWG process, NIR spectra obtained during the process were analyzed from different aspects by multivariate methods to determine the granules’ powder dynamics and API content. The results showed that the linear motion of granules in the barrel produced enough representative measurements for developing a model with a low prediction error [54].

NIR chemical imaging (NIR-CI) is a promising technique that combines traditional optical imaging and NIR spectroscopy. It is especially suitable for the measurement of particles and provides better process visualization and characterization of the TSWG. The NIR-CI image has been used as a qualitative and quantitative analytical method to characterize the mixing and flow of material in the TSWG [55]. The residence time distribution (RTD) was detected and investigated as a function of process parameters (screw rotation speed and material feeding rate) and screw configuration (number and angle of kneading elements). NIR-CI provides the possibility to better characterize and visualize the phenomenon of particle segregation along the TSG barrel and conduct process optimization to ensure granule quality [56]. The mixing behavior and the distribution of liquid and powder during the granulation process can be visualized by NIR-CI. Moisture homogeneity of granules was characterized and verified by the moisture map, which helps analyze the mixing state of liquid and powder material transportation inside the screw chamber [57].

Raman spectra is another representative molecular vibrational spectroscopic method. The application of Raman spectra requires no sample pre-treatment. The fast and non-destructive measurement of the method is suitable and used as the PAT tool. Furthermore, Raman spectra are not sensitive to water, so it is applicable for wet granulation process monitoring. An in-line API quantification method using Raman spectroscopic was developed to determine the API concentration for the TSWG process [58], indicating that Raman spectroscopy is one of the promising PAT tools for the API determination and process monitoring for the TSWG process. It should be noted that the measurement needs to be taken in the dark, as Raman spectra are influenced by light. Thus, the Raman on-line analytical method needs to be optimized to enable measurement in interior light conditions [59]. The results showed that the prediction error could be reduced significantly by optimizing the measurement setup.

### 3.2. Imaging Technique

The imaging technique showed great potential for process monitoring of the continuous TSWG. It can measure the manufacturing process in a non-contact manner with no sample consumption, saving time and cost. Furthermore, due to the fast response time, it can determine and visualize the agglomeration behavior and the state of the material in static and dynamic modes during the granulation process. Besides, this technique can provide multi-dimensional information such as particle size and distribution, shape and surface morphology, etc.

Eyecon^TM^ 3D imaging system (Innopharma Laboratories, Dublin, Ireland) is a direct imaging particle analyzer generally used for process detection for TSWG. It has a digital camera surrounded by green, red, and blue light sources. To obtain the particle’s three-dimensional (3D) features, the illumination direction of the light sources is placed following the rule of photometric stereo. The surface orientation models were calculated by transforming the image intensities on different light illuminants to detect and reconstruct the edges of the particles. An iterative algorithm was adopted to obtain the best fitting ellipse for the granules from the projected two-dimensional (2D) image and compute the equivalent area diameter.

The feasibility of implementing the Eyecon^TM^ 3D imaging system for in-line monitoring of the TSWG process was evaluated. The study showed that Eyecon™ (Innopharma Labs, Dublin, Ireland) provided good in-line images and PSD information despite the granules with a dense moving flow [60]. The capability of the Eyecon^TM^ camera for the in-line size monitoring and controlling of granules in TSWG was evaluated. Eyecon^TM^ could detect the size enlargement and count reduction when the L/S ratio changed [61]. In addition, the Eyecon^TM^ exhibited sensitivity to the perturbations and the variations in the TSWG process [62].

Besides PSD data, imaging techniques can obtain 2D properties distributions, including particle size and its distribution and liquid content of granules, to get better insight into the TSWG [34].

Imaging techniques can also obtain the temperature information of the subjects. For example, the FLIR A655sc infra-red camera, equipped with a 45° lens and a detector, can monitor the temperature of granules. When coupled with the properties of granules produced by TSWG, it can enhance overall understanding of the wetting mechanisms during TSWG [63].

### 3.3. Acoustic Emissions Technique

The acoustic emissions (AE) technique shares the advantage of NIR spectroscopy in that it is a non-invasive technique that can be utilized in real time monitor of the manufacturing process. However, instead of capturing optical signal, AE methods capture the mechanical information of process events.

The previous research has demonstrated that AE, a non-destructive ultrasonic technique, is a reliable predictor of Gaussian PSD [64,65,66,67]. The maximum amplitude of the time-domain AE signal, generated by the collision of a moving particle with a rigid surface, is correlated with particle size to estimate a single descriptor, typically d_50_. In order to enhance the ability of AE to predict a bimodal PSD for TSWG, H.A. Abdulhussain et al. adopted frequency-domain signal analysis along with artificial intelligence (AI) techniques [68]. Moreover, they developed a novel digital signal filter for the AE signal, which adjusts the signal based on impact mechanics to ensure that all particle sizes are accurately represented in the correlations. When using the filter, AE-based PAT showed exceptional predictive accuracy for near-elastic collisions, as demonstrated in a trial with lactose monohydrate granulation.

### 3.4. Multi-Technique Integrated Method

Different PAT methods with distinct functioning mechanisms can reflect varied aspects of information from the test subjects. Therefore, combined with the advantages and disadvantages of different methods, the multi-technique integrated method can comprehensively reflect the information on the material properties.

Complementary PAT tools, including Raman, NIR spectrometer, and SFV probe, were used to evaluate the feasibility of solid-state and particle size measurements for the TSWG process [15]. On the premise that the main challenge of probe fouling was solved, it proved that both Raman and NIR were suitable for monitoring the material properties of the model drug (theophylline) throughout the wet granulation process, and the SFV probe showed the ability for the in-line measurement of particle size and the particle size distribution. Furthermore, multivariate data obtained by the three PAT tools were used to monitor the granules’ moisture content, particle density, and flowability [53]. The results indicated that the moisture content showed a high degree of correlation with the NIR data, and the imaging data reflected the flowability of the granules. It has been proved that multi-technique integrated for the TSWG process can obtain complementary information.

Meng et al. [69] used three PAT methods, i.e., imaging technique, NIR, and Raman, to monitor the TSWG process. The three probes were mounted over a belt of a conveyor platform that carries on the granules from TSWG to avoid fouling or contamination of probes. For monitoring different properties, Eyecon^TM^ monitored the granule size and shape variation, NIR combined with chemometrics for physical property prediction, and Raman spectroscopy was employed to measure the content of drug components and transformations between materials. The study demonstrated the implementation of different PAT tools for continuously in-line/on-line monitoring of produced granules, which provide a better process design and monitoring of TSWG.

## 4. Process Control

According to ICH Q10 and Q13 guidelines, the quality of production could be ensured by establishing control strategies to maintain the process under control, in other words, to use process monitoring tools and control methods for the improvement of product quality, assuring the continued applicability and capability of processes [70].

QbD principle is a useful tool for establishing the control strategies for continuous manufacturing, as it provides the basic concepts and scheme for control model construction [71]. To establish variable rate process control strategies for solid oral dosage forms in continuous manufacturing, a comprehensive and systematic methodology was established for improving process understanding and control strategy for TSWG, followed by the QbD principle [72]. Based on risk assessment and DoE results, process optimization could define an appropriate operating parameter range. The empirical control strategy assures product quality consistency through model-based adjustment of process parameters in real-time, increases throughput, and reduces the incident risk of complete shutdown. As a general guideline for the control strategy of continuous manufacturing, it is useable across different unit operation chains.

Multivariate statistical process control (MSPC) strategy based on principal component analysis was a way to develop a control strategy for a continuous pharmaceutical manufacturing line. The barrel temperature, screw speed, and liquid feeding rate were detected during manufacturing for a TSWG [73]. Hotelling’s T^2^ and the corresponding Q residuals statistics control charts were applied to analyze and evaluate the impact of the fluctuations to get profound knowledge of the process. It was found that the model can monitor the performance of the manufacturing line and can detect process disturbances. In-line measurement of the granule size with the imaging camera, the particle size, and count were then assessed using the Shewhart control charts [61], providing a real-time characterization of product quality attributes for TSWG applications. Fanny Stauffer et al. [74] used the MSPC strategy to display twin-screw granulation lines’ process dynamics and deviations. The developed model can identify key points of the product within manufacturing activities. It provides a new way to rationalize the sampling strategy, design the diversion strategy, and continue process verification.

Feedback control strategy as an advanced process control (APC) solution is one way of automation in-process control method. After the monotonic increasing relation between particle size and L/S ratio, the P controller was used to carry out a real-time feedback control strategy for the TSWG process implemented via a camera equipped with a custom image analysis software firstly [75]. The liquid feeding rate was chosen as the process variable (PV) to drive the granule size to the set value. The validation of the developed system showed that the controller could compensate for the impact of interference by adjusting the liquid feeding rate, and the on-line particle size analyzer can provide the granule size with a measurement error of less than 5 µm. The model predictive control method can be implemented for TSWG process control successfully as one of the feedback control strategies. By in-line NIR spectroscopy monitoring, the L/D ratio of the wet granule based on a PLS regression model and an automatic supervisory controller was established for TSWG. When inducing artificial disturbances, it correctly follows the dynamic set point and obtains an RMSE of 0.25% *w*/*w* relative to the setpoint [76]. Torque is an important PV in the TSWG process, and the feasibility of the APC approach by adjusting the torque was investigated. The results showed little correlation between the torque and the granule size, which was unsuitable for characterizing the granule size in the APC strategy for the TSWG process. This may be due to the fact that the torque is not only related to the granule size, but also affected by the material properties such as temperature and viscosity, resulting in the change of the shear force [77].

As the utilization of PAT tools becomes more sophisticated and the development of control strategies becomes more scientific, it is feasible to incorporate real-time release testing (RTRT) methods to monitor CQAs in real-time for TSWG process. This ensures the TSWG unit can produce high-quality products that meet the downstream quality requirements with minimized intermediate testing, and improve overall operational efficiencies while ensuring product quality.

## 5. Conclusions and Future Perspectives

TSWG is a rapidly popular technology for pharmaceutical processes. However, the interaction between the screw configuration (the conveying and mixing elements), the process conditions, and the formulation properties makes the TSWG a complex process. The large diversity in the properties of raw materials, excipients, and binder liquid in different formulations diversify the products manufactured by TSWG. The influence law of these critical process variables on the critical properties of granules for TSWG process remains poorly comprehended. Although the research on TSWG has made great progress in the past few decades, there still exists considerable demand and potential for process understanding and optimization to improve the utilization of this technique. The black box approach such as the DoE method was used to understand the granulation mechanisms; mechanistic modeling tools for mechanism study can help increase the granulation mechanisms compared with the black-box approach, while the mathematical formulations of these numerical methods are extremely complex and slow when applied to practical problems, mainly due to the high computational requirements. Thus, improving the current models is necessary to monitor the continuous production process. The switch from the batch-mode of granulation process to the continuous TSWG process needs to consider how to assess and assure the drug quality and safety.

The application of PAT tools in practical production is facing many challenges. These include the reasonable layout of sensors, the way for large process data processing, multi-sensor integrated control simultaneously, etc., that need to be thoroughly considered for extensive applications. Several research studies have investigated the application of different PAT tools for monitoring of the TSWG process, including NIR spectroscopy, Raman spectroscopy, and acoustic emission techniques as mentioned in the paper. These tools have been shown to provide real-time monitoring of CQAs such as moisture content, granule size, and drug content uniformity. However, the successful application of each PAT tool depends on several factors, including the characteristics of the material, the configuration of the twin-screw granulator, and the operating conditions to ensure the measurement accuracy. Therefore, it is important to carefully assess the applicability of each PAT tool based on the characteristics of the process.

To ensure the quality of the product from CM in meeting the regulatory requirements and guidelines, accurate control strategy for each unit in the CM line including TSWG was needed to regulate the pharmaceutical process, which highlighted the importance of understanding the process. To ensure that the products from CM meet regulatory requirements and guidelines in terms of quality, an accurate control strategy for each unit in the CM line is necessary, which highlighted the importance of process understanding for TSWG. This work summarized the progress in process understanding and development for TSWG to provide a comprehensive reference for future studies.

## Figures and Tables

**Table 1:** Some examples that utilized the QbD principle to achieve process understanding and optimization on TSWG.

No.	Formula (*w*/*w*)	CPP	DoE Method	CQA for Granules	Modeling Method	Reference
1	Powder: API; povidone; hypromellose. Liquid: water	Screw speed; mill speed.	Central composite response surface statistical design	Granule chord length; particle size.	Bivariate fit	[19]
2	Powder: MCC; lactose monohydrate; starch. Liquid: HPC; HPMC; PVP.	Water-binding capacity; barrel temperature; powder feed rate; liquid addition; screw configuration.	A two-level full-factorial design.	PSD; Span; HR; bulk/tapped density; moisture content; flowability.	PLS	[20]
3	Powder: α-Lactose monohydrate. Liquid: water	Screw speed; throughput; L/S ratio; number and stagger angle of the kneading discs.	Full factorial experimental design.	RTD; mixing capacity.	PLS	[21]
4	Powder: acetaminophen;a-lactose monohydrate; MCC; PVP K 29-32. Liquid: water.	L/S ratio; throughput; rotation speed.	Face-centered cubic design.	Granule size, porosity, flowability, particle morphology.	ANOVA model	[22]
5	Powder: API; MCC; lactose monohydrate; HPMC; croscarmellose sodium; Calcium carbonate; Polysorbate 80. Liquid: water.	Screw speed; throughput; L/S ratio.	Central composite face-centered experimental design.	Torque; granule residence time; fines, yield; over-sized; volume average granule diameter; relative width; Carr Index.	Second-order polynomial models	[23]
6	Powder: Ibuprofen; dibasic calcium phosphate anhydrous. Liquid: ethanol	DCPA/Polymer ration; binder amount (%); L/S ratio.	Fractional factorial design of experiment.	Release (%), d_50_; specific surface area.	Regression analysis	[24]
7	Powder: MCC; α-lactose monohydrate; mannitol. Liquid: HPMC; PVP.	3 PCs to include the overarching properties of 8 selected pharmaceutical fillers; binder type; binder concentration.	D-optimal interaction design	Bulk/tapped density; HR; d_63.2_; fine fraction; yield fraction; coarse fraction; flowability; friability; specific surface area; torque.	Multiple linear regression	[25]
8	Powder: API; MCC; HPMC. Liquid: HPMC.	PC 1; PC 2; L/S ratio.	D-optimal screening design; D-optimal optimization design	Span; friability; fine fraction.	MLR model	[26]
9	Powder: API; Lactose monohydrate; croscarmellose Sodium; PVP K29/32. Liquid: water.	Throughput; screw speed; the screw element.	Box–Behnken experimental design	Fines; yield; over-sized; volume average granule diameter; relative width; Carr Index.	Non-linear quadratic mathematical model	[14]
10	Powder: caffeine anhydrous; α-lactose monohydrate; MCC; PVP K30. Liquid: water.	Barrel temperature; L/S ratio; throughput.	Sequential experimental strategy: D-optimal design and response surface design.	d_10_, d_50_, d_90_, Span; Eccentricity; porosity; bulk density; tapped density; HR; torque.	Stepwise least squares regression	[27]
11	Powder: APIs; MCC; HPMC. Liquid: water.	L/S ratio; screw speed.	Central composite circumscribed designs.	Fines; yield; HR; oversized; tapped density; friability; torque.	Quadratic polynomial models	[28]
12	Powder: metformin hydrochloride; mebendazole; MCC; α-lactose monohydrate; HPMC. Liquid: water.	Fraction of KE in the first; kneading zone; KE thickness; screw speed; throughput; L/S ratio.	D-optimal design	Yield; over-sized; fines; bulk density; HR; friability.	MLR models	[29]
13	Powder: Ibuprofen; MCC; lactose monohydrate; Croscarmellose sodium; Hydroxypropyl cellulose; Colloidal silicon dioxide. Liquid: water.	L/S; throughput; screw speed; screw configuration; barrel temperature.	A full factorial design	The angle of repose; bulk/tapped density; PSD; granule strength; granule morphology.	Forward stepwise regression	[30]
14	Powder: (1) lactose; PVP. (2) lactose; HPMC. (3) lactose; MCC; PVP. (4) lactose; MCC; HPMC. Liquid: water.	Nozzle diameter; nozzle orientation; throughput; screw speed; screw configuration; barrel temperature; L/S ratio; total binder content; the relative fraction of binder added dry.	Plackett–Burman design for screening.	Oversized; fines; HR; bulk density; tapped density; yield; the angle of repose, torque.	Linear models	[31]
15	Powder: metformin hydrochloride; α-Lactose monohydrate; MCC; HPMC. Liquid: water.	3 PCs to include the MCC properties; L/S ratio; screw speed; drying time.	Two-level full factorial design.	Fines; HR; torque; LOD.	MLR	[32]
16	Powder: lactose monohydrate; MCC; PVP K25. Liquid: water.	L/S; SFL; screw length; DFS	Two-level full factorial design.	RTD; particle Size; shape distributions.	Multiple linear regression	[33]
17	Powder: MCC; α-lactose monohydrate Liquid: PVP.	Liquid feed rate; powder feed rate; L/S ratio; screw speed; viscosity.	Full factorial DoE.	RTD; PSD; liquid content distribution.		[34]
18	Powder: lactose; copovidone, PVP, hyprolose, hypromellose Liquid: water.	L/S; SFL; screw speed; powder feed rate.	3^2^ experimental design and single-factor experiment.	PSD; span.	-	[35]

Microcrystalline cellulose (MCC); polyvinylpyrollidone (PVP); hydroxypropyl methylcellulose (HPMC); water binding capacity (WBC); Hausner ratio (HR); kneading elements (KE); length to diameter (L/D); residence time distribution (RTD); high specific feed load (SFL); distributive feed screw (DFS); partial least squares (PLS); particle size distribution (PSD); hydroxypropyl cellulose (HPC); multiple linear regression (MLR); analysis of variance (ANOVA); loss on dry (LOD).

## Data Availability

Not applicable.
